# Formation of large area closely packed carbon onions film by plasma-based ion implantation

**DOI:** 10.1038/s41598-020-67323-9

**Published:** 2020-06-22

**Authors:** Naohiro Matsumoto, Hiroshi Kinoshita, Junho Choi, Takahisa Kato

**Affiliations:** 1 Department of Mechanical Engineering, Graduate School of Engineering, University of Hyogo, 2167, Shosya, Himeji, Hyogo 671-2280 Japan; 2 Department of Mechanical Engineering, The University of Tokyo, 7-3-1, Hongo, Bunkyo-ku, Tokyo 113-8656 Japan

**Keywords:** Engineering, Materials science, Nanoscience and technology

## Abstract

A substantial quantity of carbon onions in a durable film state is indispensable for its applications. In this study, large area fabrication of closely packed homogeneous carbon onion nanoparticle film using plasma-based ion implantation was demonstrated. Ag film deposited on a Si substrate was used as the implantation target for the hydrocarbon ions accelerated at 20 kV. Nanoparticles with the mean diameter of 7.5 nm were formed at the grain boundary of the Ag film. Carbon onions with the mean diameter of 17.4 nm were synthesized and arranged to a closely packed nanoparticle film with the thickness of around 200 nm by gradual thermal vaporization of the Ag. The closely packed configuration was achieved due to the isolated growth of carbon onion nanoparticle and high uniformity of the diameter. This process can be used in principle large area formation compered to typical ion implantation technique of carbon onion nanoparticle film, which can be applicable for the practical use in mechanical and electrochemical applications.

## Introduction

Carbon onion, which is a member of nanocarbon family, has an “onion” like structure, namely a sphere consisting of multilayered-concentric-graphitic shells^1^. The typical diameter of the carbon onion is in the range of 5–100 nm, which has been expected to possess unique mechanical and electrical properties such as high strength, low friction and high conductivity because of its characteristic graphite-based microstructure and spherical shape. The production of carbon onions in a film state of large area and high-particle density is considered to be a key technology to realize the practical applications of carbon onions in wide-industrial fields such as batteries, solid lubricants, protective coatings, solar cells, and data storage devices^2–8^. Similar to other nanocarbon materials such as carbon nanotubes or carbon nanohorns^9,10^, various methods have been proposed to synthesize carbon onions^1,11–13^. The structure of carbon onions was first discovered in carbon soot irradiated with a dense electron beam by Ugarte^1^. Following that, the syntheses from diamond nanoparticles by annealing or electron irradiation were reported, which is one of the typical methods for recent fundamental research on carbon onions^14–17^. Carbon onions can also be formed by arc-discharge of graphite in water^12^. However, the practical application of carbon onions is not so much progressed due to the limitation in the homogeneous production of nanoparticle and the film formation of carbon onions.

An ion implantation technique to synthesize carbon onions, which has a potential to fabricate a carbon onions in film state, was firstly reported by Cabioc'h et al.^13^, where mass-selected pure carbon ions were implanted into noble metals such as Cu or Ag with the acceleration energy of more than 100 kV. Plasma-based ion implantation (PBII) technique is widely-used for the surface coating and modification of common metal components^18–20^. In comparison with the conventional implantation technique, where high-energy acceleration and mass-selection mechanisms are equipped, the PBII adopts a simple implantation process such that ions in the plasma are directly implanted with relatively low energy into the substrate without any mass-selections of ions.

In this study, we applied PBII technique to synthesize carbon onions with methane as the plasma source. C-ions were irradiated to an Ag film as an implantation target. The synthesis process of carbon onions film consists of three steps: Ag film deposition, C-ions implantation, and Ag film evaporation. The stability of the density and the translational energy of the ions in the plasma in PBII is less than that in conventional implantation process, although the implantation area in principle is not limited because the acceleration electric field covers for the entire substrate surface^21^. This implantation mechanism has an advantage on the formation of large-area and three-dimensional carbon onions film on various component surface, which can contribute not only to reveal the basic characteristics of carbon onions, but also to realize the practical applications such in electrical, mechanical, and optical devices. The formation process of carbon onions film was analyzed by using transmission electron microscopy (TEM) and X-ray photoelectron spectroscopy (XPS). The configuration of the nanoparticle film was observed by field emission scanning electron microscopy (FE-SEM). Tribological and mechanical properties of the carbon onions film were evaluated.

## Experiments

### Formation of nanoparticle film

The fabrication of carbon onions film consists of mainly three processes: Ag film deposition, carbon ion implantation, and elimination of the Ag on the substrate. Ag films were deposited on a Si(100) substrate under room temperature by electron–cyclotron-resonance sputtering (Elionix, Inc.) of the Ag target by Ar ions. The flow rate and the pressure of Ar gas introduced in the chamber were fixed at 10 sccm, and 10^–2^ Pa, respectively, and the acceleration voltage of Ar was at 2 kV. The deposition rate of the Ag film was quantified to 8 nm/min, and the thickness of the Ag film was measured to approximately 250 nm by the cross-sectional images of FE-SEM (Hitachi, S4800). The average size of the sputtered Ag grains of the film was estimated to 26 nm before PBII and 40 nm after PBII using full width of the half maximum (FWHM) of the Ag(111) X-ray diffraction (XRD) (Rigaku, SmartLab) peak by Scherrer's equation (SI Fig. S1).

The ion implantations into the Ag film were conducted using the PBII facility (Kurita Industry). The detail of the facility was described elsewhere^20^. The base pressure of the vacuum chamber was kept to 10^–4^ Pa. Methane gas was used as a plasma source with a flow rate of 5 sccm and a pressure of 0.1 Pa. In PBII process, dual positive and negative pulsed biases were applied to the substrate alternatively at the frequency of 4 kHz. In this study, 0.5 kV of the positive and 20 kV of the negative pulses were applied to the substrate. The pulse time of 5 μs was used for both the positive and the negative bias voltages, and the delay time of the negative pulse subsequent to the positive was for 20 μs. The implantation duration was 10 min.

Ag evaporation was conducted by using plasma heating. Applying a positive bias voltage to the substrate, electrons in the plasma were accelerated to the substrate and the substrate temperature was increased. Ar gas was introduced and the pressure was kept at 0.05 Pa. The positive bias voltage of 10 kV was applied to the substrate, and the temperature was kept at 900 °C for 5 h.

Ag film after the implantation and carbon onions particles film were analyzed by SEM, TEM and XPS. The specimens for the cross-sectional TEM (JEOL, JEM-2000EXII) observations were prepared by mechanical cutting, followed by hand polishing and ion-beam polishing. Prior to the TEM observations, the surface of the samples was protected with a thermosetting-epoxy resin. TEM observations were performed at an acceleration voltage of 200 kV in this study. The depth profiles of the chemical structure of the carbon C1s in Ag film were analyzed by XPS by 300 W AlKα with 0.2 kV Ar sputtering. The cross-sectional SEM observations of the nanoparticle films were performed by cleaving the Si(100) substrate.

### Nanoparticle film properties measurements

Mechanical property of the synthesized carbon onions film was measured by Nanoindentation using a Berkovich diamond indenter. The hardness and elastic modulus were evaluated by Oliver and Pharr method^22^. Indentation loads of 10, 100, and 1,000 μN were used for the measurements. Friction forces of the nanoparticle film were measured by using AFM under the normal loads of 0–120 nN.

## Results and discussion

**Figure 1:**
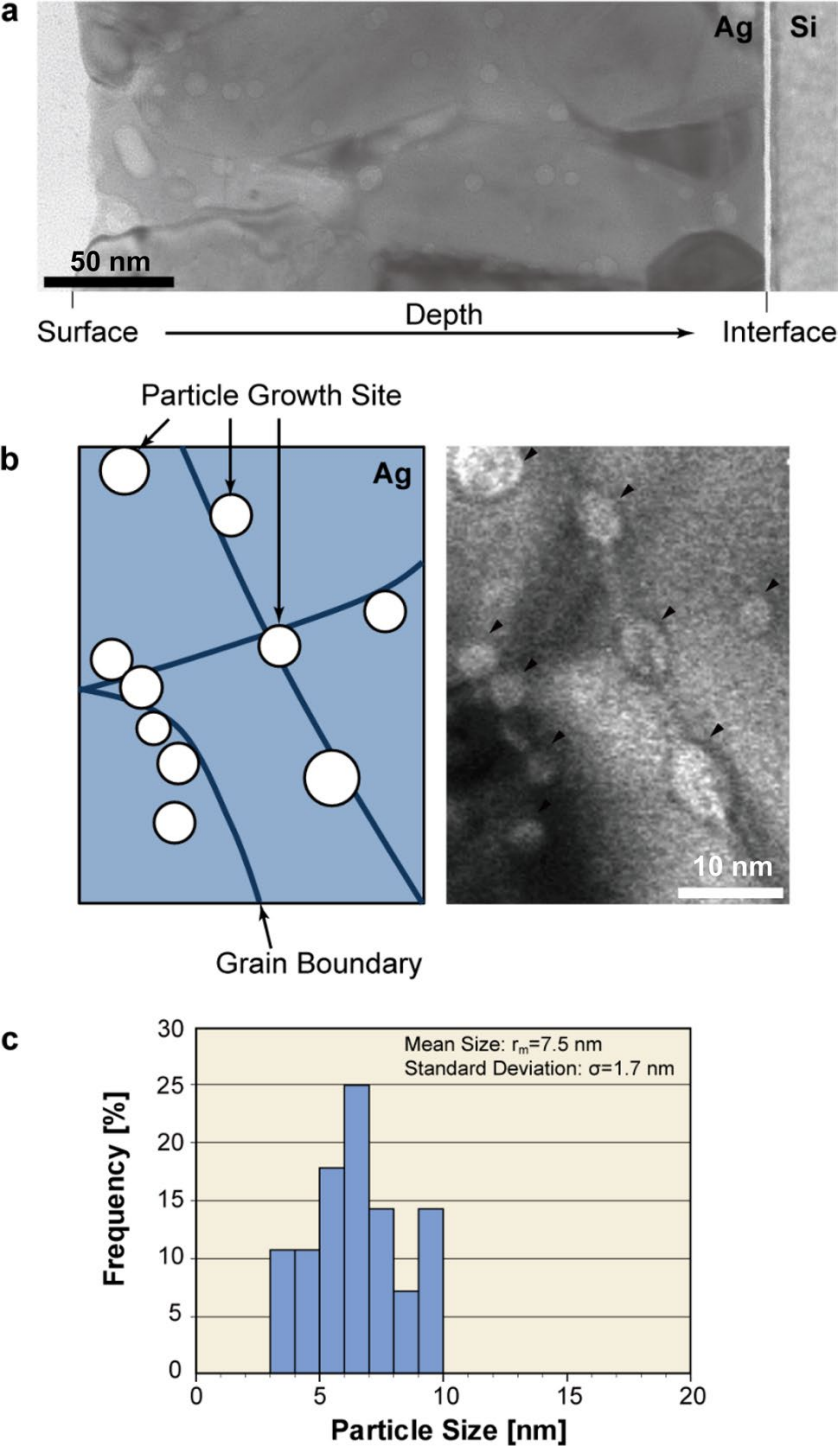
(Color online) Carbon nanoparticle formation in Ag films. (**a**) Cross-sectional TEM image of the Ag film after methane plasma implantation of carbon ions accelerated at 20 kV. The ions were implanted from the surface (left) to the interface (right). (**b**) Magnified TEM micrograph of (**a**). Bright spots indicated by arrows denote the carbon nanoparticle formation. (**c**) The size distribution of the nanoparticles of (**a**).

Figure 1 shows the methane-plasma implanted Ag film observed by TEM. The cross-sectional image of the Ag film is shown in Fig. 1a, where the implantation was directed from left to right. It can be seen that bright-circular spots were dispersed in the whole sectional area of the Ag film of the thickness of 250 nm, which are considered to be segregation of the implanted-carbon atoms in the Ag matrix^23^. From the detailed observation of the magnified TEM image and the corresponding illustration in Fig. 1b, segregation of the carbon atoms took place mainly along the grain boundaries of the Ag grains. The statistical analysis in Fig. 1c showed that the mean and the standard deviation of the spot sizes were determined to 7.5 nm and 1.7 nm, respectively. Hence, the size of the segregated nanoparticles shows a sharp distribution in the Ag film with this method.

**Figure 2:**
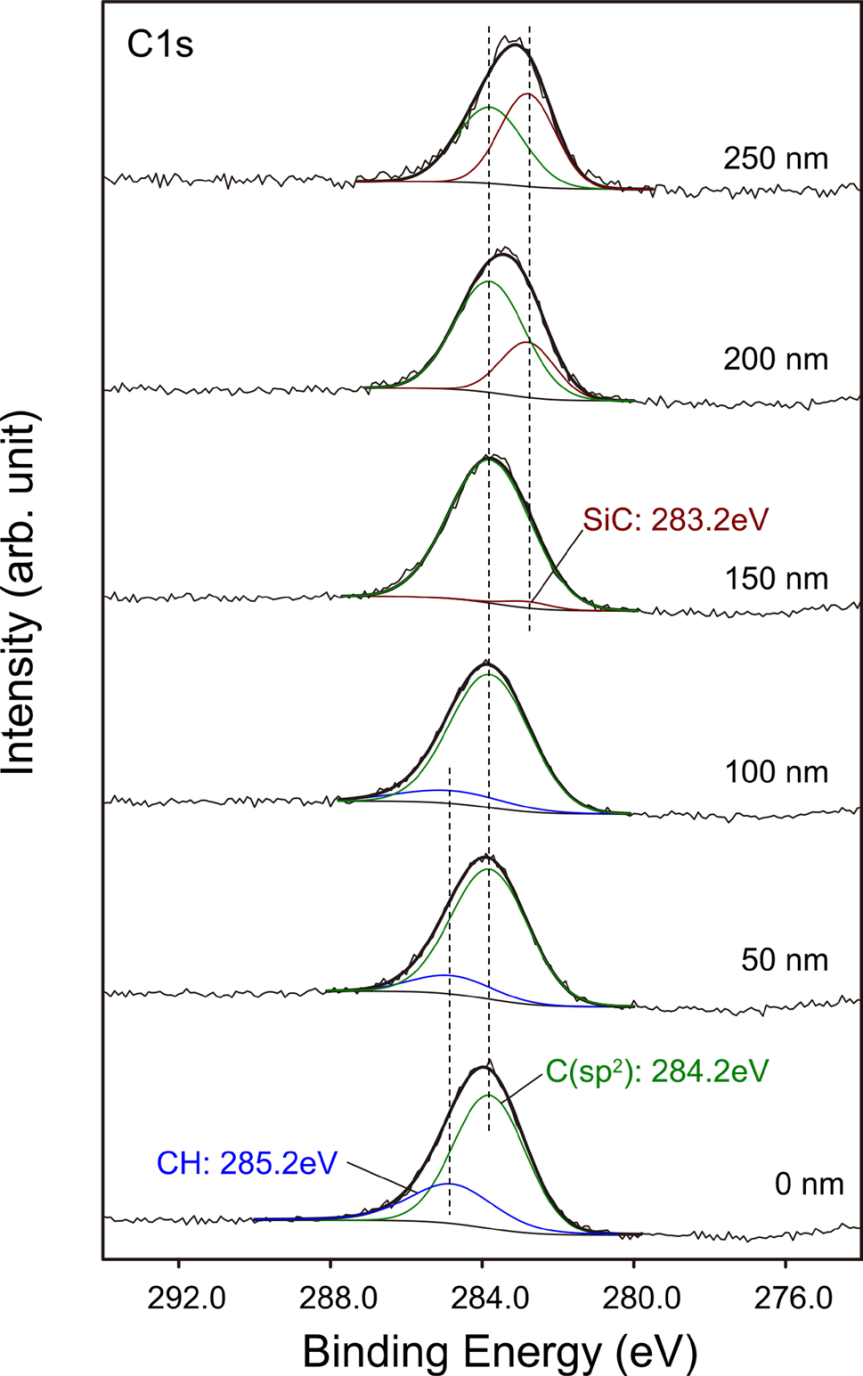
(Color online) C1s XPS core spectra of the methane plasma implanted silver surface at the depth of 0, 50, 100, 150, 200, and 250 nm.

In the present PBII process, the implanted ions consist of various fragments of methane such as CH^+^, CH_2_^+^, and etc. The inclusion of hydrogen in the ions is likely to affect the nucleation and crystallization of carbon onions. Figure 2 shows C1s XPS core spectra of the silver surface after the hydrocarbon ions implantation. The intensity of CH bond at 285.2 eV gradually decreased and the C (sp2) bond at 284.2 eV increased as deeper the silver surface. Also, around the interface between silver and silicon substrate, the peak from SiC bond emerged from the depth more than 150 nm. That is the implanted hydrocarbon ions loose hydrogen as increasing the penetration depth into the silver. It can be explained by the catalytic effect of the metal on the dissociation of the CH bond. The isolated hydrogen might re-evaporate from the silver surface or stored in the interstitial of the silver. In contrast, apparent peak shift with the depth was not observed for Ag3d spectra (SI Fig. S2).

**Figure 3:**
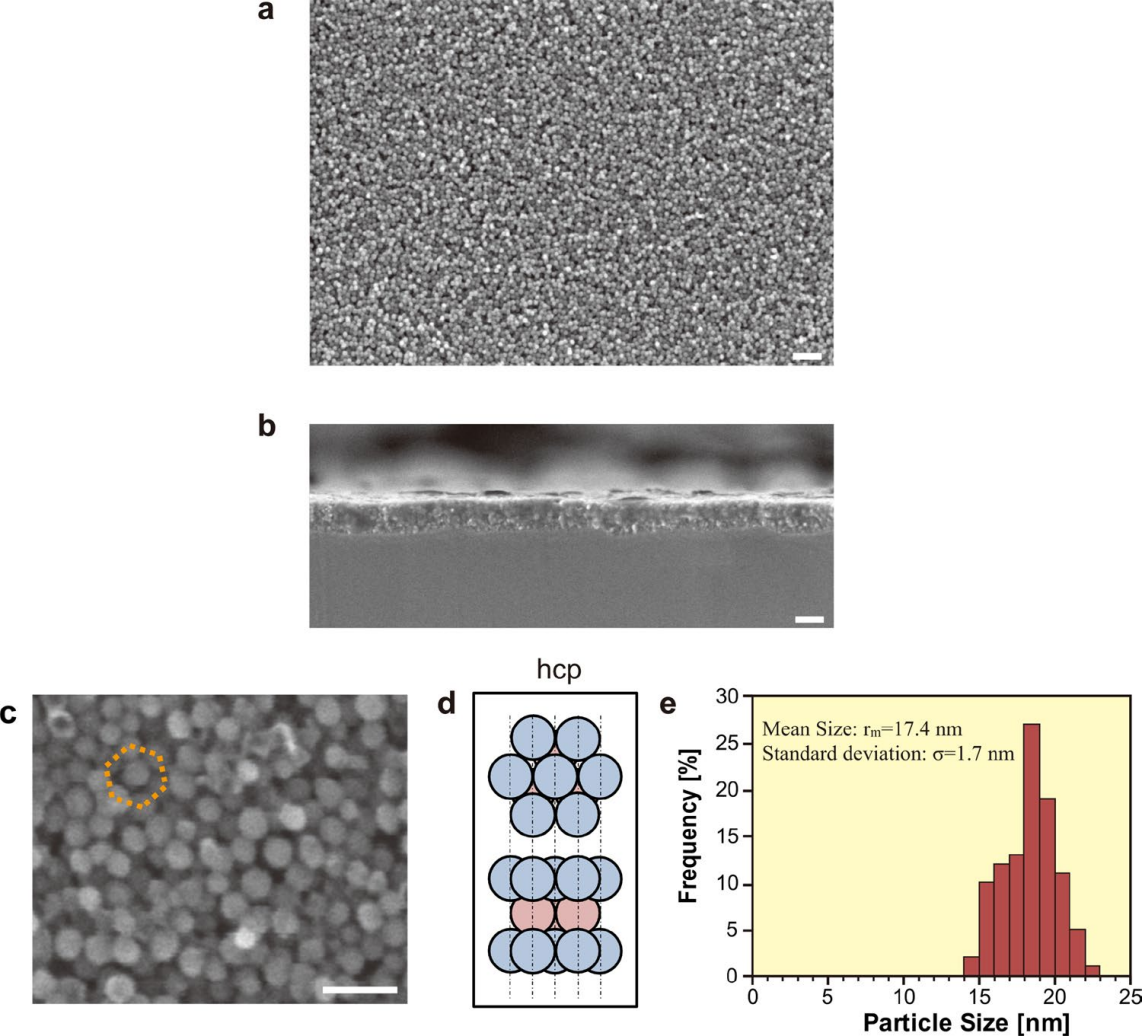
(Color online) Closely packed homogeneous carbon nanoparticle films. (**a**) Top view of the film observed by FE-SEM. (**b**) Sectional view of the film. (**c**) Magnified FE-SEM micrograph of (**a**). (**d**) Schematic of hcp alignment. (**e**) The size distribution of the carbon nanoparticles of (**a**).

The carbon-implanted Ag film was then heated in vacuum to evaporate and eliminate the Ag atoms from the film to obtain carbon nanoparticle film. Figure 3 shows the nanoparticle films obtained after Ag elimination heated at 900 °C for 5 h. SEM images of the top and side views of the nanoparticle film after the heat treatment are shown in Fig. 3a,b. The formation of carbon nanoparticle film was seen on the whole Si substrate as shown in Fig. 3a with a uniform thickness of around 200 nm in Fig. 3b. A magnified image of the top view of Fig. 3c shows that the carbon nanoparticles were closely packed configuration in the nanoparticle film. Moreover, some of the carbon nanoparticles are arranged in an almost hexagonal close-packed (hcp) arrangement, as denoted by the regular hexagon in Fig. 3c and illustrated in Fig. 3d. The statistical analysis in Fig. 3e showed the sharp distribution of the diameter of carbon nanoparticles; the average and standard deviation of the diameter were 17.4 nm and 1.7 nm, respectively. An energy-dispersive X-ray spectrometry (EDX) analysis confirmed non-existence of Ag atoms after the heat treatment, that is, pure carbon nanoparticles left on the Si substrate (SI Fig. S3).

**Figure 4:**
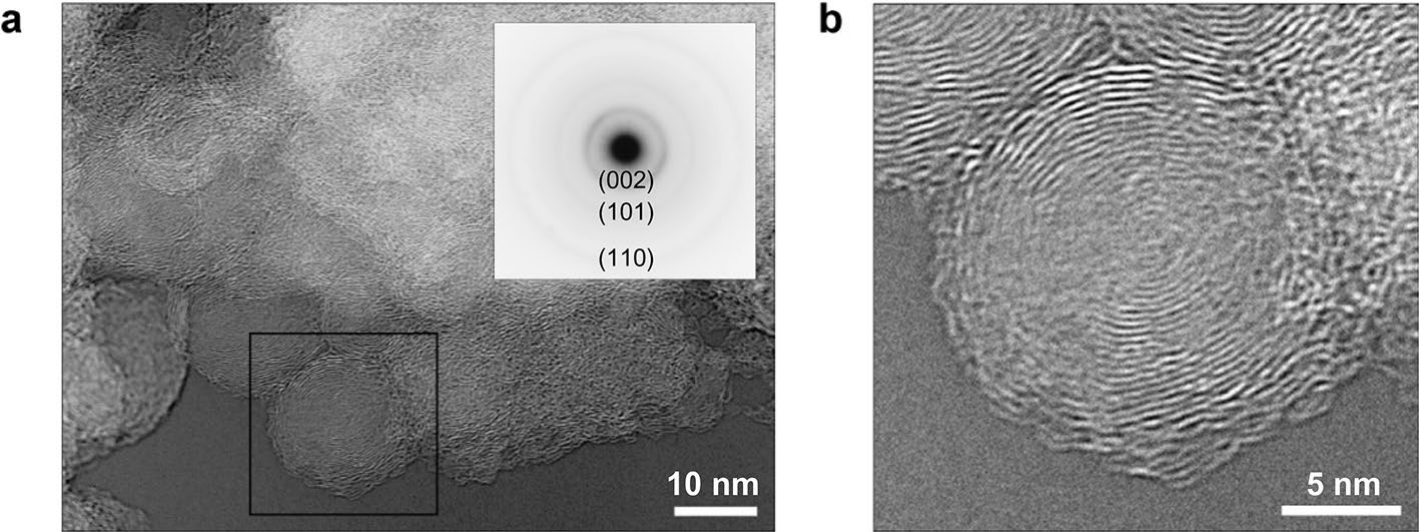
TEM images of nanoparticles formed by the elimination of Ag. (**a**) Agglomerated carbon onions. An electron diffraction pattern is shown in the inset. (**b**) Magnified TEM image of the area denoted by the square in (**a**).

Figure 4 shows the microstructure of the carbon nanoparticles observed by TEM. The bright field images of Fig. 4a and magnified Fig. 4b demonstrate that each carbon nanoparticle has a multilayered spherical shell structure, namely the carbon onion structure. The diffraction ring from graphite (002) is clearly observed as show in Fig. 4a. The distance between the layers is 0.35 nm, which is close to that of graphite 0.34 nm.

**Figure 5:**
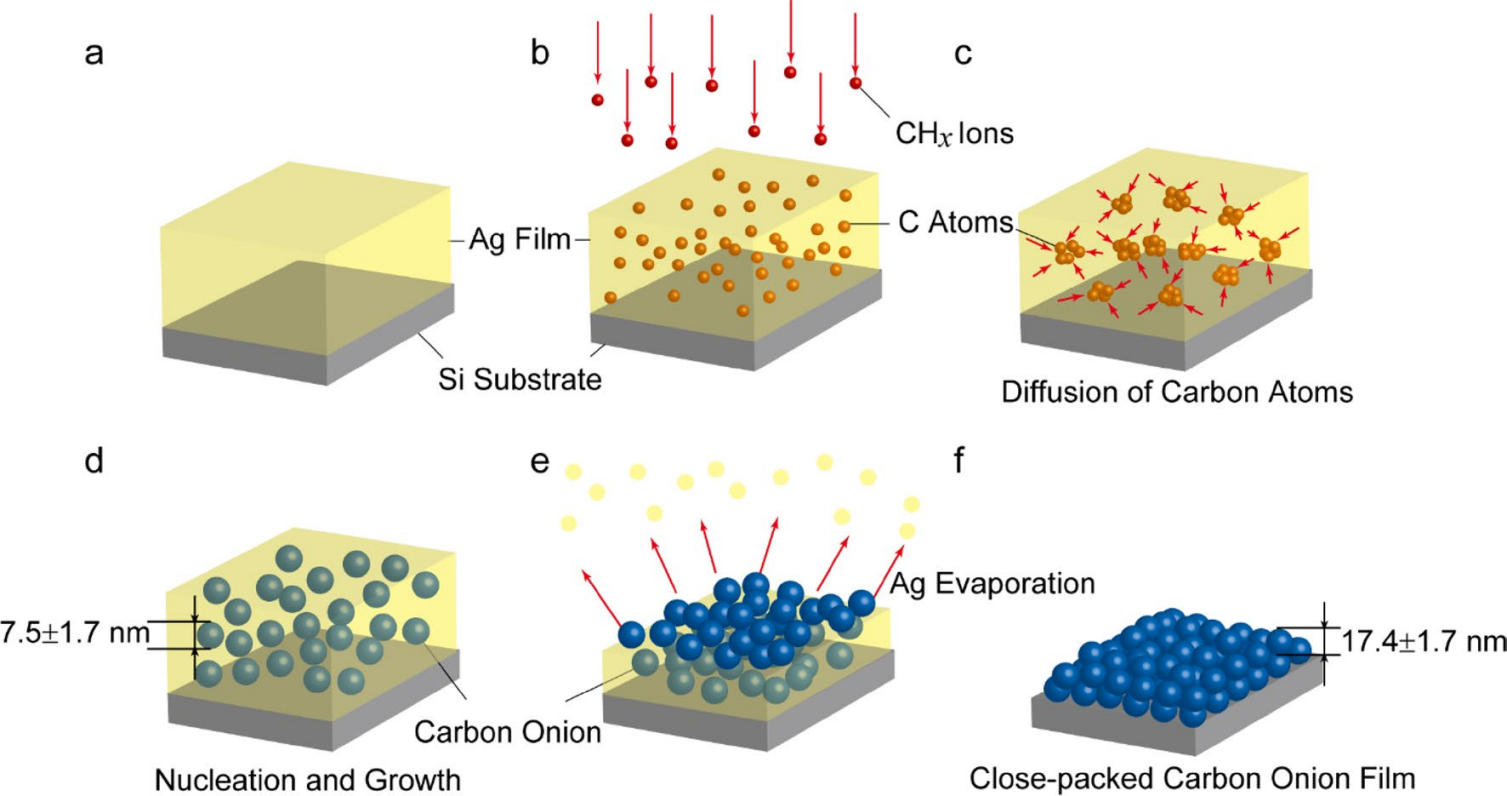
(Color online) Summary of the formation process of carbon onions film. (**a**) Polycrystalline Ag film deposited on a Si substrate. (**b**) Carbon ion implantation into the Ag film. (**c**) Carbon ion diffusion in the Ag film. (**d**) Nucleation of carbon onions. (**e**) Elimination of Ag by vacuum heating. (**f**) Formation of the closely packed homogeneous carbon onions film.

The fabrication process of the closely-packed-homogeneous carbon onions film is summarized in Fig. 5. On Ag film deposited on Si substrate (Fig. 5a), implanted carbon ions impact on the Ag atoms in the film until they lose their translational energy as Fig. 5b, and diffuse at high temperature toward energetically stable sites as Fig. 5c, which are mostly the Ag grain boundaries. Therefore, the carbon ion density becomes enormously high at the grain boundaries where they seek a more stable state, namely the nucleation of carbon onions as Fig. 5d. In the progress of the elimination of Ag atoms in the final step, the mono-dispersed carbon onions grow and approach one another in the film as Fig. 5e. It was observed that the carbon onions grew from the average diameter of 7.5 nm, as shown in Fig. 1c, to 17.4 nm, as shown in Fig. 3e, during the thermal heating at 900 °C after the ion implantation. Therefore, we can say that the size of the carbon onions and their density are strongly affected by Ag grain size and the thermal process. Finally, the closely packed state of carbon onions is obtained where nanoparticles of carbon onions are attached to each other inside the film, and attached to the Si substrate as Fig. 5f. It is noted that a high implantation energy, e.g. more than 100 kV, was considered necessary for the formation of carbon onions, as reported in previous works^11,13^. The present study, on the other hand, showed that carbon onions were nucleated with implantation energy of 20 kV, and grew at around the evaporation temperature of the Ag atoms. The high particle density can be achieved by the isolated formation of the microstructure of carbon onions because it can prevent the chemical bonding between nanoparticles which restrict the movement of the nanoparticles to the hexagonal stable position.

To synthesize a large amount of carbon onions throughout the substrate, carbon ions must be supplied throughout the entire Ag film. Hence, a broad distribution of carbon ions through the depth of the Ag film is required. The broad distribution of carbon ions in this study is mainly due to the polycrystalline structure of the Ag film. The implantation depth of the carbon ions into single crystalline Ag can be calculated by using conventional transport of ions in matter (TRIM) code^24^, and the attainable depth of C^+^ ions in single crystalline Ag is about 80 nm at an acceleration voltage of 20 kV. The observation by cross-sectional TEM, on the other hand, revealed that the carbon ions arrived at the interface between the Ag film and the Si substrate as shown in Fig. 1a, namely, the maximum implantation depth of carbon ions was more than 250 nm. The longer reaching depth of the carbon ions compared with the calculated implantation depth for single crystalline Ag is attributed to the polycrystalline structure of the sputter-coated Ag film, and more particularly to the grain boundaries, where the Ag atomic density is small, resulting in a smaller suppression force against the ion implantation compared with the single crystalline structure. It is also considered that the implantation method of PBII affects the carbon ions distribution in the Ag film. In PBII, the potential energy of carbon ions generated in the plasma ion sheath, which is formed along the substrate surface, varies with the distance from the substrate surface^25^. The implantation energies of the carbon ions are not uniform, and neither are the reaching depths of the carbon ions in this process. In addition, the heating induced by the ion implantation itself activates the thermal diffusion of the carbon ions in the Ag film.

**Figure 6:**
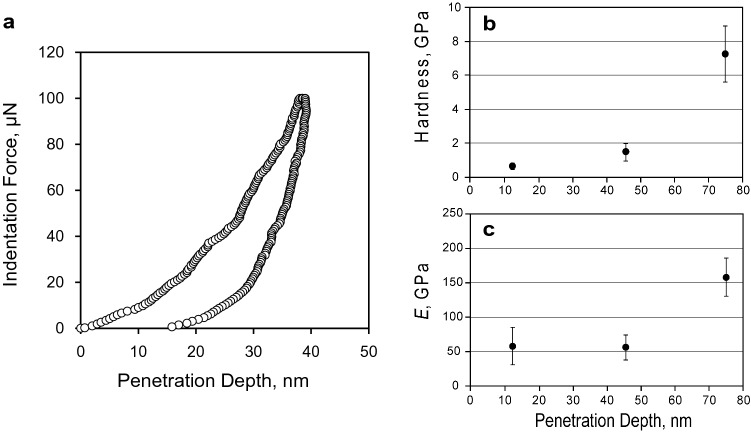
Nanoindentation tests results of the carbon onion nanoparticle films. (**a**) Typical load–displacement curves of the nanoindentation tests. (**b**) Indentation hardness and (**c**) elastic modulus E of the nanoparticle film with the penetration depth.

A wide range of applications of the presented nanoparticle film can be anticipated due to high density of the carbon onions. In tribological applications, a low frictional property of carbon onions on molecular scale measured by atomic force microscope (AFM)^26^ and in liquid lubricant as a nanoparticle additive^27^ is reported. The high degree of the sphericity and the low surface energy of the graphite-base surface would attribute to the low frictional property of carbon onion. In fact, the carbon onions were arranged to close-packed film state, which is explained by the low interactive energy between nanoparticles. The indentation tests were conducted by the penetration depth of less than 80 nm for the nanoparticle film with the thickness of around 200 nm. Figure 6 shows mechanical property measurements of the carbon onion nanoparticle film by using nanoindentation. Continuous indentation curves were obtained as shown in Fig. 6a in all measurements. Indentation hardness and elastic modulus E with the penetration depth are shown in Fig. 6b,c. Drastic increases were observed at the penetration depth around 80 nm for both hardness and E due to the effect of Si substrate. The hardness and E of Si substrate are known to around 15 and 160 GPa, which is higher than the nanoparticle film measured in this study. When the interfacial strength between relatively soft nanoparticle film and hard Si substrate is enough large not to be delamination, a compressive pressure is produced between the substrate and diamond indenter under indentation, which cause the increase of the measured hardness and elastic modulus of the soft film^28^. The intrinsic hardness and E of the nanoparticle film was evaluated around 2 GPa and 50 GPa respectively, in which the indentation hardness is comparable to bulk copper^29^, at the penetration depth of around 40 nm before the drastic increase of the hardness and E to avoid the influence of Si substrate. At the indentation load of 1,000 μN, which is maximum load we used, continuous load–displacement curve was obtained (SI Fig. S4), which means no fracture of the nanoparticle film including delamination of the nanoparticle film at the interface between film and Si substrate. In addition, the drastic increase of the hardness with increasing the penetration depth is affected by the strong adhesion of the film to the substrate^28^. Therefore, the adhesive property of the nanoparticle film is thought to be not weak. These adhesive properties of nanoparticle film were obtained by the formation of SiC chemical bonding between nanoparticle film and Si substrate as shown by XPS in Fig. 2.

**Figure 7:**
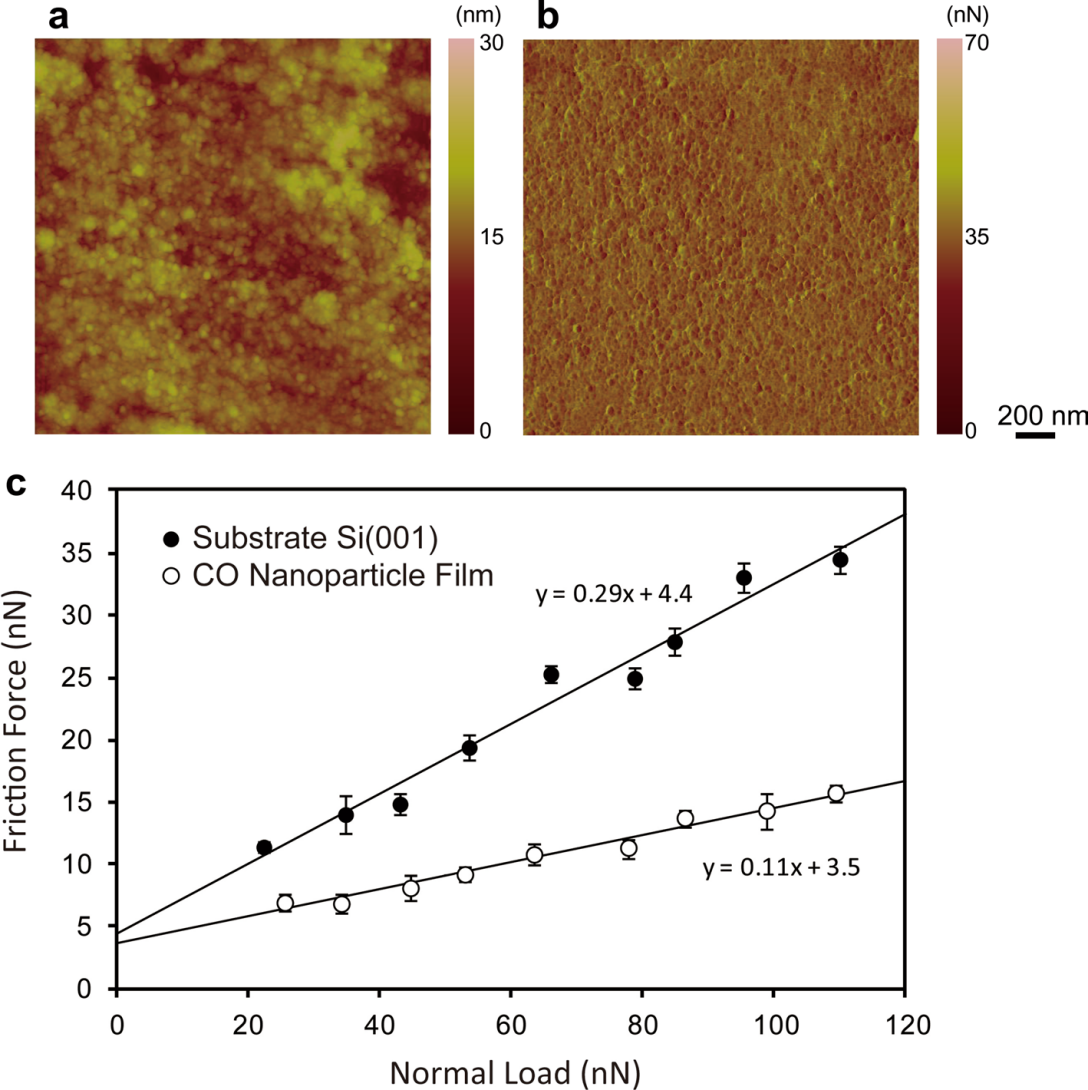
(Color online) Tribological property measurement of the nanoparticle film by AFM. (**a**) The height and (**b**) lateral force images of the nanoparticle film. (**c**) Friction force measurements results of the nanoparticle film and bare Si substrate.

Figure 7 shows the tribological property measurement of the nanoparticle film by AFM. From the height and lateral force images shown in Fig. 7a,b, individual nanoparticles were observed and almost uniform lateral forces on the nanoparticle film were detected. Friction forces were evaluated under the normal loads of 0–120 nN. The measured friction forces for the nanoparticle film and bare Si substrate were shown in Fig. 7c. Friction forces of the nanoparticle film was proportional to the normal load as well as the bare Si substrate, and the friction coefficients were evaluated to 0.11 and 0.29, respectively. Therefore, it can be said that the prepared nanoparticle film has good lubricating property with moderate durability.

Closely-packed configuration of the carbon onion nanoparticle film would have advantages in properties such as higher surface area, density, and durability compared to the general loosely-packed carbon onions nanoparticle film. These properties can be suitable for various applications. Carbon onions has been applied as an electrode for an electrochemical supercapacitor because of a high specific surface area and high electrical conductivity of carbon onion^2^. So far, the carbon onion-based electrochemical electrode was prepared with chemical binder which causes the degradation of the power of discharge. One of the possible applications of the present carbon onions film can be electrochemical applications.

## Conclusions

A fabrication of a closely packed homogeneous carbon onion nanoparticle film was presented. The method consists of three steps; Ag film deposition on the Si substrate, methane plasma ion implantation into Ag film and thermal vaporization of Ag. It was shown that the homogeneous carbon onions with the uniform diameter of 17.4 nm were formed with a partially hexagonal closely packed alignment by means of the controlled thermal evaporation of Ag. The high particle density can be achieved by the isolated formation of the microstructure of carbon onions because it prevented the formation of chemical bonding between nanoparticles which may restrict the movement of the nanoparticles to the hexagonal stable position. The prepared nanoparticle film shows good self-lubricating properties with moderate durability.

## Supplementary information

Supplementary file1 (DOCX 145 kb)
