# Supplementary material for: Formation of large area closely packed carbon onions film by plasma-based ion implantation
Source: Sci Rep. 2020 Jun 22;10:10037. doi: 10.1038/s41598-020-67323-9 (PMC7308266; doi:10.1038/s41598-020-67323-9)
Supplement: Supplementary file 1 — Supplementary file1 (DOCX 145 kb) [file 41598_2020_67323_MOESM1_ESM.docx]

Supplementary Information

Formation of large area closely packed carbon onions film

by plasma-based ion implantation

Naohiro Matsumoto^1)^ *, Hiroshi Kinoshita^1)^, Junho Choi^2)^, and Takahisa Kato^2)^

^1)^ Department of Mechanical Engineering, Graduate School of Engineering, University of Hyogo

2167, Shosya, Himeji, Hyogo 671-2280, Japan

^2)^ Department of Mechanical Engineering, The University of Tokyo

7-3-1, Hongo, Bunkyo-ku, Tokyo 113-8656, Japan

*Corresponding author: matsumoto@eng.u-hyogo.ac.jp


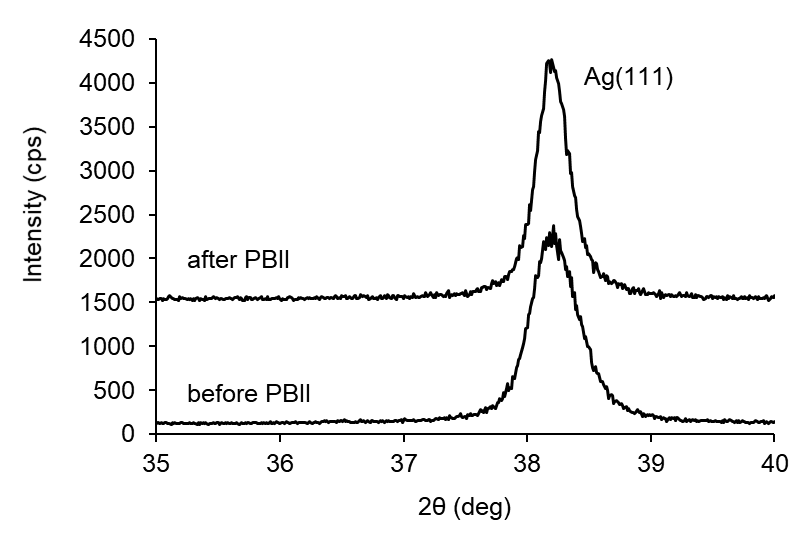


Figure S1 X-ray diffraction spectrum of the sputtered Ag film on Si substrate by electron-cyclotron-resonance sputtering before and after PBII. The average size of the Ag grains were estimated to 26 nm before PBII and 40 nm after PBII from the Ag(111) peak.


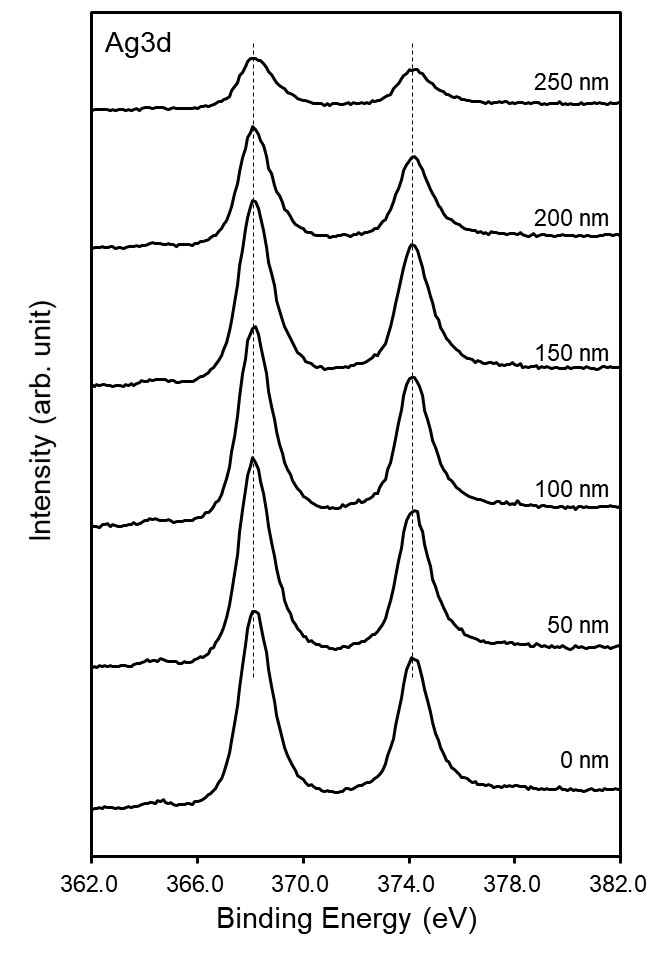


Figure S2 Ag3d XPS core spectra of the methane plasma implanted silver surface at the depth of 0, 50, 100, 150, 200, and 250 nm.


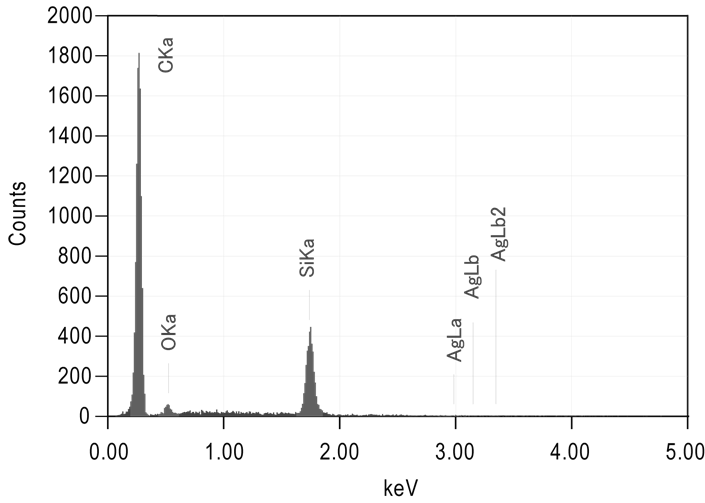


Figure S3 EDX data for the nanoparticles film obtained by the heat treatment of the carbon-implanted Ag film


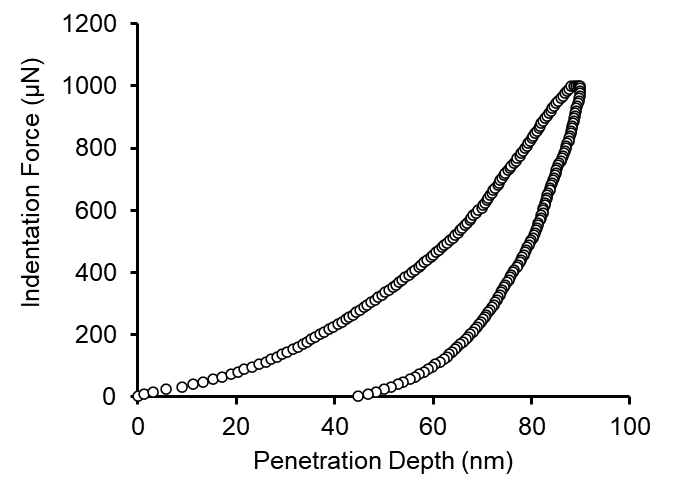


Figure S4 Indentation force vs penetration depth curve measured by the indentation load at 1000 μN
